# Daily Medication Management and Adherence in the Polymedicated Elderly: A Cross-Sectional Study in Portugal

**DOI:** 10.3390/ijerph17010200

**Published:** 2019-12-27

**Authors:** Daniel Gomes, Ana Isabel Placido, Rita Mó, João Lindo Simões, Odete Amaral, Isabel Fernandes, Fátima Lima, Manuel Morgado, Adolfo Figueiras, Maria Teresa Herdeiro, Fátima Roque

**Affiliations:** 1 Research Unit for Inland Development—Polytechnic of Guarda (UDI-IPG), 6300 Guarda, Portugal; danielsgomes98@hotmail.com (D.G.); anaplacido@ipg.pt (A.I.P.); isabelfernandes@ipg.pt (I.F.); mmorgado@ipg.pt (M.M.); 2 Centre for Health Studies and Research of the University of Coimbra, 3000 Coimbra, Portugal; 3 Health Sciences Faculty, University of Beira Interior (FCS-UBI), 6200 Covilhã, Portugal; ritamdmo@hotmail.com; 4 Center for Health Technology and Services Research (CINTESIS), 4000 Porto, Portugal; jflindo@ua.pt; 5 Health Sciences School, Polytechnic of Viseu IPV, 3430 Viseu, Portugal; mopamaral@gmail.com; 6 Local Health Unit of Guarda (ULS Guarda), 6300 Guarda, Portugal; dir.csaudeprimarios@ulsguarda.min-saude.pt; 7 Pharmaceutical Services, University Hospital Center of Cova da Beira (CHUCB), 6200 Covilhã, Portugal; 8 Health Sciences Research Centre, University of Beira Interior (CICS-UBI), 6200 Covilhã, Portugal; 9 Department of Preventive Medicine, University of Santiago de Compostela, 15702 Santiago de Compostela, Spain; adolfo.figueiras@usc.es; 10 Centro de Investigación Biomédica en Red de Epidemiología y Salud Pública (CIBER-ESP), 28001 Madrid, Spain; 11 Department of Medical Sciences and Institute of Biomedicine, University of Aveiro (iBIMED-UA), 3800 Aveiro, Portugal; teresaherdeiro@ua.pt

**Keywords:** elderly, polypharmacy, daily management of medication, medication adherence, self-care, nonadherence related factors

## Abstract

The presence of age-related comorbidities prone elderly patients to the phenomenon of polypharmacy and consequently to a higher risk of nonadherence. Thus, this paper aims to characterize the medication consumption profile and explore the relationship of beliefs and daily medication management on medication adherence by home-dwelling polymedicated elderly people. A questionnaire on adherence, managing, and beliefs of medicines was applied to polymedicated patients with ≥65 years old, in primary care centers of the central region of Portugal. Of the 1089 participants, 47.7% were considered nonadherent. Forgetfulness (38.8%), difficulties in managing medication (14.3%), concerns with side effects (10.7%), and the price of medication (9.2%) were pointed as relevant medication nonadherence-related factors. It was observed that patients who had difficulties managing medicines, common forgetfulness, concerns with side effects, doubting the need for the medication, considered prices expensive, and had a lack of trust for some medicines had a higher risk of being nonadherent. This study provides relevant information concerning the daily routine and management of medicines that can be useful to the development of educational strategies to promote health literacy and improve medication adherence in polymedicated home-dwelling elderly.

## 1. Introduction

According to the United Nations (UN), the number of elderly (≥65 years) will double until 2050 [1]. One of the reasons explaining the worldwide ageing of population is the exponential technological contributions in the medicine and public health field, leading to an increase in life expectancy and decrease of mortality rate [2,3]. Medicines are the medical technology mostly used and, according to literature, the concomitant use of five or more medicines, the most widely accepted definition of polypharmacy, affects 40–50% of elderly in high-income countries [4,5,6,7]. The use of multiple medication contributes to an incorrect use of medicines by the elderly and may potentially increase the risk for a Drug Related Problems (DRPs) occurrence, therefore interfering with the treatment effectiveness and safety [8,9,10]. In this context, DRPs in the elderly account for a large percentage of emergency treatment and hospitalizations, increasing the costs with health in the most aged regions [11,12,13]. In addition, levels of adherence to drug therapy correlate inversely with the number of drugs prescribed, especially in the elderly who do not live in nursing homes [8,14].

Adherence in the elderly can be compromised, either by volunteering interrupt or modify the therapy, by mistakenly believing that they are properly adhering, or ascribed to some relevant socio-economic factors, elder’s perceptions, motivations and physical and cognitive impairment, their own condition, and even the complexity of the therapy [14,15,16]. 

Since Portugal is one of the most aged countries in the world and the center region of Portugal is the second region with the highest aging index in the country [17,18], we designed the MedElderly project, which the main goal is the identification of DRPs in the older patients and the development of an intervention to improve medication use by this population. In this paper, we present the results of the first phase of the project that aim to characterize the medication profile consumption, assess medication adherence, and identify relevant risk factors affecting adherence in home-dwelling older patients of the center region of Portugal.

## 2. Materials and Methods 

### 2.1. Setting, Study Population, and Sample Selection

A cross-sectional study was conducted in 38 public primary care centers between April and August 2019, belonging NUTS II (Nomenclatura das Unidades Territoriais para Fins Estatísticos/Nomenclature of Territorial Units for Statistics) area of Portugal defined by the Center Regional Health Administration (Administração Regional de Saúde do Centro/ARS-C). Primary care health centers of ARS-C are organized in nine clusters. The study took place in five clusters with 227,785 elderly patients registered. The target population included home-dwelling patients 65 years or older taking five or more medicines. Individuals with cognitive impairment or mental conditions that prevented them from responding appropriately and those who lived in nursing homes were excluded from the study. The sample population of the study was calculated based on the number of elderly patients that are registered in primary care centers and an estimated prevalence of elderly with medication misadventures of 30% keeping a 95% confidence level and a 5% tolerated error level. Considering a possible refusal rate of 20%, the final sample size was calculated to be 387 older patients. 

### 2.2. Questionnaire Design

To assess medication adherence and risk factors of medication misadventures in a target population, a questionnaire was designed taking into account data from two qualitative studies with focus-group sessions held with older patients [17] and healthcare professionals (physicians, community pharmacists, and nurses) [18]. 

To ensure that the questionnaire was easily understood, well-defined, and accurately addressed the goals of the study, one clinical psychology expert, one general practitioner, two pharmacists, two nurses, and one Portuguese language expert evaluated face-validity parameters, such as the accuracy, the grammar, syntax, organization, appropriateness and logical sequence of the statements, and completeness and meaning of items. Moreover, to clarify possible problems of comprehension with any questionnaire item, a pre-test was conducted, eleven older adults were invited to complete the questionnaire and comment on any difficulties experienced in interpreting the respective items [19].

### 2.3. Data Collection

Healthcare professionals screened potential candidates for participation in each health care center, nursing and pharmacy students from four-health science institutions, approached potential participants before or after the scheduled visit to the doctor’s appointment, for questionnaire application, confirmed their eligibility, and invited them to participate in the study. Participation in the study was voluntary, and all subjects received a detailed explanation about the goals, objectives, methods, and the purpose of the study. An application manual of the questionnaire and a training course were developed for the students who would apply it. 

The structured questionnaire was 5 sheets long, 7 pages, divided in seven sections: Section 1, containing information about the study, and instructions on how to complete the form and a box with information about the interviewers. The second section comprises multiple choice questions about daily administration of medications by patients, and the third section comprises affirmations about the elder’s opinion about medicines, asking which ones he/she agreed with (yes or no question). The therapeutic adherence was evaluated in the fourth section with the “Medida de Adesão aos Tratamentos” (MAT) scale, by Delgado et al. [20], validated for the Portuguese population. This instrument consists of 7-items rated on a six-point Likert scale ranging from “Always” to “Never”, and in all questions “Always” is the lowest adherence point and “Never” is the highest. The fifth section consists of 10-item scales rated on a four-point Likert scale (plus No answer) concerning situations that may sometimes contribute to not taking the medication exactly as prescribed, ranging from “Never happens” to “Happens almost every time”. The sixth section concerns the sociodemographic characteristics of the participants and the last section intends to list all the chronic medication, extracted from the electronic records, then converted to the corresponding Anatomical Therapeutic Classification (ATC) code, using the WHO Collaborating Centre for Drug Statistics Methodology’s web site [21].

### 2.4. Statistical Analysis

Data analysis was executed using the Statistical Package for Social Sciences (SPSS 25, IBM Corp., New York, NY, USA) and all *p* values ≤ 0.05 were considered statistically significant. Numerical and ordinal data were analyzed using descriptive statistics and presented in frequency and percentage and using mean, median, and standard deviation. The “Don’t know/don’t answer” situation was addressed and as considered missed values. 

To measure the internal consistency of the applied scale, Cronbach’s α was used. Cronbach′s α can range from 0.0 to 1.0, and it quantifies the degree to which items on an instrument are correlated with one another. 

Regarding the MAT scale, the adherence level from each individual was obtained summing up the values from the seven questions and dividing the value by the number of questions. The classification as adherent and nonadherent used the median as the cut value. Below the median, the sample is nonadherent and, above it, is adherent. To clear the results, whenever the adherence classification was involved, the sample within the median value was excluded [20].

The variables showed not to be normal (*p* < 0.001) so, for searching associations, non-parametric tests were used. For quantifying associations between two variables, continuous or ordinal, the Spearman Correlation value was obtained. For searching for risk factors interfering with adherence classification, multiple regression analyses were performed. 

### 2.5. Ethics Statement

The project had the ethical approval of the Centre’s Regional Health Administration (registry no. 105/2017) and the authorization of the healthcare centers directors. The participants granted informed consent prior to the administration of the questionnaire. The participants were codified and remained anonymous in the database. 

## 3. Results

### 3.1. Sample Characterization

Table 1 presents the sociodemographic characteristics of the sample. The mean (± SD) age of the 1089 participants was 76.2 (± 7.07), and 62.6% were women. In addition, 60.8% of the participants lived with a partner, 27.3% lived alone, and 8.6% with sons and/or grandchildren. Moreover, 76.5% of the participants had an education level of elementary school or higher, 7.7% did not know how to read or write, and 15.8% did know but without any degree. In addition, 58.8% of the participants had a monthly income less than 439 EUR. The mean of medication prescription was 6.82 ± 2.03, with a median of six medicines, and ranged from 5 (*n* = 348) to 17 (*n* = 1).

### 3.2. Medication Consumption Profile

Within anatomical groups of the ATC, the group A “Alimentary tract and metabolism” (1.32 ± 1.07; min. 0; max. 6), group C “Cardiovascular system” (2.50 ± 1.40; min. 0; max. 10), and group N “Nervous system” (1.32 ± 1.29; min. 0; max. 8) were most frequent. Table 2 presents the frequency of individuals of the sample consuming medicines from the pharmacological Groups A, C, and N of ATC.

Drugs for peptic ulcer and gastro-oesophageal reflux disease (46.4%), blood glucose lowering drugs, excl. Insulins (37.3%), beta blocking agents (26.7%), lipid modifying agents (59.7%), anxiolytics (38.6%), and antidepressants (22.4%) were highlighted as the most frequent. 

### 3.3. Medication Adherence Analysis

According to Cronbach’s α, the MAT scale showed a satisfactory intern consistency (α = 0.767). The mean value of adherence level was 5.47 ± 0.47, and the minimum and maximum values obtained were 3 and 6, respectively (Table 3). In addition, 47.7% of the sample had an adherence value below the median and were categorized as nonadherent.

The single-question analysis of MAT scale revealed that (Table 4): the compliance with the medication schedule (second question) had the lowest mean (4.99 ± 0.82), and the fifth question, about taking one or more pills for the illness on their own after feeling worse had the highest mean (5.8 ± 0.54). Except for “Have you ever forgotten to take the medicines for your illness?” and “Have you ever been careless about the time you take your medicines?”, the majority of answers was in the “Never”, Likert scale point, ranged from 71.1% to 86.0% in the sixth question, regarding therapy interruption due to running out of medicines, and the fifth, regarding if the elderly takes more medicines when he/she feels worst, respectively. The medians from the third to the seventh questions had the maximum value possible (6.0). The frequencies were low in the three worst grades of the Likert scale, the highest 3.1% in the answer being “Often” in the first and second question.

### 3.4. Medication Nonadherence Related Factors

The most frequented medication nonadherence related factors pointed out by the elderly were the forgetfulness (38.8%), difficulties in managing medication (14.3%), concerns with side effects (10.7%), and the price of medication (9.2%) showed in Table 5.

Table 6 and Table 7 presented a multiple regression analyses to identify factors associated with medication adherence in the participants. They revealed a positive correlation between the adherence levels and age and the number of medicines prescribed (Table 6). 

Moreover, the values of Odd ratio (Table 7) revealed that patients who had: (a) difficulties managing medicines (OR = 1.720, confidence interval 95%, CL 1.189–2.489); (b) forgetfulness (OR = 3.370, confidence interval 95%,CI 2.572–4.400); (c) concerns with side effects, (OR = 3.555, confidence interval 95%, CI 2.248–5.626); (d) doubt the need for the medication (OR = 3.910, confidence interval 95%, CI 1.668–9.165); (e) prices expensive (OR = 2.290, confidence interval 95%, CI 1.455–3.605); and (f) lack of trust for some medicines (OR = 5.090, confidence interval 95%, CI 1.711–15.163) had a higher risk of being non-adherent. Elderly with a monthly income less than 439 EUR (OR = 1.701, confidence interval 95%, CI 1.314–2.203) had an increased probability of being non-adherent. 

### 3.5. Daily Medication Management

Despite 82.7% of the participants being responsible for the managing of their medicine, 74.6% of the participants admitted that did not know the name of their medicines and recognized them only by: (a) the box (56.7%), (b) the color of the pills (12.8%), or (c) the shape of the pills (5.1%). The participants admitted that, in the morning (fasting, 9.3% and breakfast, 10.4%) and lunch (10.7%) sometimes, they had difficulty in remembering to take their medicines. To avoid forgetfulness, the majority of the participants used a medication box (39.5%), put the pills in different bags/places according to the schedule that the medicines must be taken (23.3%) and at the beginning of the meal put the medicines on the table (20.8%). Participants admitted that, in daily life, they had difficulties with schedules (14.6%), swallowing the pills (13.6%), remembering how they should take its medicines (4.8%), and 2.1% affirmed that they had difficulties with respiratory devices, such as inhalators.

## 4. Discussion

According to our results, almost half of the polymedicated elderlies were nonadherent. In our study, nonadherence was attributed to forgetfulness, difficulties in managing medication, concerns with side effects, the price of medication, doubt about the need for the medication and the lack of trust for some medicines, showing that elderly’s behaviors, beliefs, and attitudes have an impact on medication adherence.

Adherence to medication is fundamental to achieve clinical outcomes and ensure the improvement of the health being of elderly. It is well known that medication adherence is influenced by several external factors, namely psychological status of the patient and by the patient community [14]; however, there is a lack of studies regarding how the opinion and daily management of medicines by elderly people influence medication adherence.

Nonadherence to medication leads to a lack of treatment effectiveness, increased hospital admissions, healthcare expenditures, and ultimately can lead to an overtreatment of a disease [22,23]. Due to the presence of multiple comorbidities that require multiple therapies, and, consequently polypharmacy, elderlies are more prone to compliance problems and nonadherence than younger [8,14]. In our study, a positive correlation between the adherence levels and age was found. Despite that, according to literature, age is not a predicative factor of nonadherence to medication [24,25]; in fact, the elderly may choose, intentionally not take medication by several reasons, namely because they don’t like medicines or to avoid adverse side effects [25]. The analysis of the medication profile of the participants revealed that drugs belonging to the ATC groups “Alimentary tract and metabolism”, “Cardiovascular system”, and “Nervous system” were the most prescribed drugs. These groups of drugs are frequently associated to an increased prevalence of suffering from drug-related problems [26,27,28].

According with Morris and Schulz [29], nonadherence to medication occurs due to the influence of friendship and family on the clinical status of the patient and can range from 40% to 75% in the elderly [30,31]. Literature reviews pointed out that, in developed countries, adherence to medication averages 50% [32,33,34] and approximately half of the disease is intentional.

Adherence to medication can also be influenced by factors such as the ability to read and understand medication instructions [35,36]. In our study, despite the fact that 23.5% of the population does not have a grade primary school and almost 60% had only an elementary school, no correlation was observed between adherence and scholar literacy [37]. 

The income (receiving less than 439 EUR) has been found to be a risk factor into nonadherence. Higher income may promote higher adherence values. This is an important result considering the fact that 58.8% of our sample receives less than 439 EUR. Related to this, it is well known that poor medication adherence increases health care costs both for the National Health Service and for the elderly, worsening the income’s problem [38].

Moreover, considering that income and education are so strongly correlated [39], it is not wrong to assume that the level of education might as well influence the adherence levels. 

Some studies suggested that gender, personality, and cultural factors could also influence adherence [40]. In our study, the marital status and living alone did not had any correlation with adherence, which is in accordance with other studies [32,39,40]. 

Given that 74.6% of the participants did not know the name of their medicines, and some participants admitted that had difficulties in managing medicines, the use of strategies to the correct management of medicines and to improvement of adherence is crucial [41]. In fact, almost 40% of participants admitted that use a medication box and a huge percentage of participants admitted that put the medicines according with its daily life, e.g., put the pills in different bags/places according with the schedule that the medicines must be taken and at the beginning of the meal put the medicines on the table. 

This study reinforces other research suggesting that nonadherence can be intentional or non-intentional. Non-intentional nonadherence is caused by forgetfulness, poor understanding of how or when to use medication, and this type of nonadherence can be avoided by daily routine strategies. Factors such as the lack of ability to recognize medicines and erroneous beliefs may increase nonadherence [7]. To improve medication adherence, it is essential to develop patient-centered strategies, namely physicians must understand why patients use medication incorrectly before prescribing medicines. Moreover, healthcare professionals should address patient beliefs and other factors such the fear of medicines.

Although it fills a gap in the literature by addressing daily management of medicines by elderly and adherence-related factors, this study had some limitations. The questionnaire was applied by different interviewers; however, in order to ensure the trustworthiness of the questionnaire, the questionnaire was developed after a qualitative study, a pretest was done, and the interviewers received training and a manual of applicability. Even though the sample was not randomized, the information obtained in this study is relevant and very useful to the field.

Within the MedElderly project, with the information obtained in this cross-sectional study, an intervention to improve medicines’ use consisting of educational sessions and educational materials (posters and flyers) was designed addressing the major DRPs identified in the targeted population. Educational material is delivered in community pharmacies and public primary health care centers. For improving the management of medication adherence, the material includes colorful pictograms to be placed by the pharmacist in the medicine boxes. 

## 5. Conclusions

Forgetfulness, difficulties in managing medication, concerns with side effects, and the price of medication were found to be significant medication management and adherence-related factors. Patients who had difficulties managing medicines, common forgetfulness, concerns with side effects, doubt of the need for the medication, considered prices expensive, and lack of trust for some medicines had a higher risk of being nonadherent. This study provides relevant information concerning the daily medication management that can be useful to develop educational strategies to promote health literacy and improve medication management and self-care for polymedicated home-dwelling elderly.

## Figures and Tables

**Table 1:** Socio-demographic characteristics of the participants.

*N* = 1089	% (*N*)
**Sex**	
Female	62.6% (680)
Male	37.4% (407)
**Age**	
(65–68)	11.2% (121)
(68–71)	15.1% (163)
(71–74)	13.1% (141)
(74–77)	15.8% (170)
(77–80)	11.8% (127)
(80–83)	12.0% (130)
(83–86)	10.4% (112)
(86–89)	6.0% (65)
(89–92)	2.9% (31)
(92–95)	1.1% (12)
(95–99)	0.6% (7)
**Monthly Income**	
<439 EUR	58.8% (579)
440–580 EUR	23.6% (232)
581–1160 EUR	14.7% (145)
≥1161 EUR	2.9% (29)
**With Who Do You Live?**	
Alone	27.0% (294)
Partner	60.1% (654)
Son and/or Grandchildren	8.5% (93)
Others	3.3% (35)
**Level of Education**	
Does not read or write	7.7% (83)
Knows how to read/write but no grade	15.8% (171)
Primary School	57.7% (626)
2° Cycle (5th and 6th grade)	7.0% (76)
3° Cycle (7th to 9th)	4.8% (52)
High School (10th to 12th)	3.5% (38)
Medium grade	0.8% (9)
Higher Education/Graduate	2.7% (29)
**Region Living**	
Interior	70.5% (768)
Coast	29.5% (321)

**Table 2:** Consumption of medicines from the fourth level pharmacological groups of Group A, Group C, and Group N.

	% (*N*)
Group A	
A01A stomatological preparations	0.2% (2)
A02A antacids	0.3% (3)
A02B drugs for peptic ulcer and gastro-oesophageal reflux disease	46.4% (501)
A03A drugs for functional gastrointestinal disorders	1.2% (13)
A03B belladonna and derivatives, plain	0.3% (3)
A03F propulsives	2.1% (23)
A05A bile therapy	0.1% (1)
A06A drugs for constipation	1.3% (14)
A07A intestinal antiinfectives	0.1% (1)
A07D antipropulsives	0.3% (3)
A07E intestinal antiinflammatory agents	0.7% (8)
A07F antidiarrheal microorganisms	0.2% (2)
A09A digestives, incl. Enzymes	0.4% (4)
A10A insulins and analogues	7.6% (83)
A10B blood glucose lowering drugs, excl. Insulins	37.3% (403)
A11A multivitamins, combinations	0.1% (1)
A11C vitamin a and d, incl. Combinations of the two	4.8% (52)
A11D vitamin b1, plain and in combination with vitamin b6 and b12	1.7% (18)
A11E vitamin b-complex, incl. Combinations	0.3% (3)
A11G ascorbic acid (vitamin c), incl. Combinations	0.1% (1)
A12A calcium	6.4% (69)
A12C other mineral supplements	1.6% (17)
A13A tonics	0.1% (1)
Group C	
C01A cardiac glycosides	2.3% (25)
C01B antiarrhythmics, class i and iii	3.9% (42)
C01D vasodilators used in cardiac diseases	4.9% (53)
C01E other cardiac preparations	5.8% (62)
C02A antiadrenergic agents, centrally acting	1.9% (20)
C02C antiadrenergic agents, peripherally acting	0.2% (2)
C03A low-ceiling diuretics, thiazides	0.2% (2)
C03B low-ceiling diuretics, excl. Thiazides	9.2% (99)
C03C high-ceiling diuretics	18.7% (201)
C03D potassium-sparing agents	3.2% (35)
C03E diuretics and potassium-sparing agents in combination	1.6% (17)
C04A peripheral vasodilators	3.2% (35)
C05A agents for treatment of hemorrhoids and anal fissures for topical use	0.2% (2)
C05B antivaricose therapy	0.6% (6)
C05C capillary stabilizing agents	7.2% (77)
C07A beta blocking agents	26.7% (289)
C07B beta blocking agents and thiazides	0.2% (2)
C07C beta blocking agents and other diuretics	0.1% (1)
C08C selective calcium channel blockers with mainly vascular effects	14.8% (159)
C08D selective calcium channel blockers with direct cardiac effects	1.9% (20)
C08G calcium channel blockers and diuretics	0.1% (1)
C09A angiotensin-converting-enzyme inhibitors, plain	16.4% (176)
C09B angiotensin-converting-enzyme inhibitors, combinations	14.5% (156)
C09C angiotensin II receptor blockers, plain	18.3% (198)
C09D angiotensin II receptor blockers, combinations	21.3% (230)
C09X other agents acting on the renin-angiotensin system	0.2% (2)
C10A lipid modifying agents, plain	59.7% (644)
C10B lipid modifying agents, combinations	3.5% (38)
Group N	
N01A anesthetics, general	0.2% (2)
N02A opioids	8.2% (88)
N02B other analgesics and antipyretics	13.2% (143)
N02C antimigraine preparations	0.1% (1)
N03A antiepileptics	9.3% (100)
N04B dopaminergic agents	2.9% (31)
N05A antipsychotics	4.5% (48)
N05B anxiolytics	38.6% (406)
N05C hypnotics and sedatives	4.6% (50)
N06A antidepressants	22.4% (242)
N06B psychostimulants, agents used for adhd and nootropics	2.6% (28)
N06D anti-dementia drugs	4.3% (46)
N07A parasympathomimetics	0.1% (1)
N07C antivertigo preparations	8.1% (87)
N07X other nervous system drugs	0.1% (1)

**Table 3:** Minimum, maximum, mean and median of the adherence levels.

	Minimum	Maximum	Mean ± SD	Median
**Adherence Level**	300 (*N* = 2; 0.2%)	6.00 (*N* = 167; 15.4%)	5.47 ± 0.47	5.57

**Table 4:** Results from the Questions of Adherence Treatment Measure (MAT) and respective Mean and Median.

	Always % (*N*)	Almost Always % (*N*)	Often % (*N*)	Sometimes % (*N*)	Seldom % (*N*)	Never % (*N*)	Mean ± SD	Median
1. Have you ever forgotten to take the medicines for your illness?	0.1% (1)	0.4% (4)	3.1% (34)	20.1% (219)	45.4% (494)	30.9% (336)	5.03 ± 0.83	5
2. Have you ever been careless about the time you take your medicines?	0.0% (0)	0.7% (8)	3.1% (34)	20.7% (225)	47.8% (521)	27.6% (301)	4.99 ± 0.82	5
3. Have you ever stopped taking medicines for your illness because you felt better?	0.0% (0)	0.2% (2)	1.7% (19)	8.2% (89)	11.8% (128)	78.1% (850)	5.66 ± 0.72	6
4. Have you ever stopped taking the medicines for your illness on your own after feeling worse?	0.1% (1)	0.3% (3)	1.6% (17)	10.2% (111)	12.9% (140)	75.0% (814)	5.60 ± 0.77	6
5. Have you ever taken one or more pills for your illness on your own after feeling worse?	0.0% (0)	0.1% (1)	0.8% (9)	3.8% (41)	9.3% (101)	86.0% (937)	5.80 ± 0.54	6
6. Have you ever interrupted therapy for your illness because you have run out of medicines?	0.0% (0)	0.2% (2)	1.0% (11)	8.9% (97)	18.8% (205)	71.1% (774)	5.60 ± 0.71	6
7. Have you ever stopped taking your medicines for some reason other than doctor′s appointment?	0.0% (0)	0.2% (2)	0.9% (10)	9.9% (108)	12.6% (137)	76.4% (832)	5.64 ± 0.7	6

**Table 5:** Frequencies on the reasons to not take the medication as prescribed.

Sometimes You Do Not Take Your Medication Exactly as Your Doctor Has Prescribed Because:	% (*N*)
a. Has difficulty managing so many medicines.	14.3% (156)
b. Forgetfulness.	38.8% (423)
c. Do not register as taking instructions.	4.4% (48)
d. Concern with side effects.	10.7% (116)
e. Hard to take.	4.8% (52)
f. Doubt the need for the medication	2.9% (32)
g. Price of medicines	9.2% (100)
h. Not wanting to take medication and drink alcohol	2.2% (24)
i. Not liking to take medications	4.1% (45)
j. Do not trust some medications	2.1% (23)
k. Interfere with social life	3.3% (36)

**Table 6:** Spearman correlation between adherence level and age and medicines prescribed.

		Coefficient Value	Significance (*p* Value)
**Adherence Levels**	Age	0.077	0.018
	Medicines Prescribed	0.082	0.031

**Table 7:** Factors influencing medication nonadherence.

Independent Dichotomous Variables	OR [95% CI] *	Χ^2^ (*p*-Value)
Sometimes you do not take your medication exactly as your doctor has prescribed because:		
Has difficulty managing so many medicines	1.720 [95% CI 1.189–2.489]	0.004
Forgetfulness	3.370 [95% CI 2.572–4.400]	<0.001
Do not register as taking instructions	1.552 [95% CI 0.835–2.885]	0.161
Concern with side effects	3.555 [95% CI 2.248–5.626]	<0.001
Hard to take	1.717 [95% CI 0.919–3.208]	0.087
Doubt the need for the medication	3.910 [95% CI 1.668–9.165]	0.001
Price of medicines expensive	2.290 [95% CI 1.455–3.605]	<0.001
Not wanting to take medication and drink alcohol	0.379 [95% CI 0.148–0.969]	0.036
Not liking to take medications	0.815 [95% CI 0.430–1.522]	0.520
Do not trust some medications	5.090 [95% CI 1.711–15.163]	0.001
Interfere with social life	1.328 [95% CI 0.661–2.667]	0.424
Sociodemographic characteristics		
Female	1.408 [95% CI 1.082–1.833]	0.011
Male	0.710 [95% CI 0.546–0.924]	0.011
Living alone	1.187 [95% CI 0.892–1.579]	0.241
Not having level of education	1.196 [95% CI 0.889–1.608]	0.237
Living in the Interior Region of Centre Portugal	2.506 [95% CI 1.874–3.351]	<0.001
Earning <439 EUR	1.701 [95% CI 1.314–2.203]	<0.001

* CI—Confidence Interval.
